# The Flares of Low back pain with Activity Research Study (FLAReS): study protocol for a case-crossover study nested within a cohort study

**DOI:** 10.1186/s12891-022-05281-1

**Published:** 2022-04-21

**Authors:** Pradeep Suri, Adrienne D. Tanus, Nikki Torres, Andrew Timmons, Bianca Irimia, Janna L. Friedly, Anna Korpak, Clinton Daniels, Daniel Morelli, Paul W. Hodges, Nathalia Costa, Melissa A. Day, Patrick J. Heagerty, Mark P. Jensen

**Affiliations:** 1 Seattle Epidemiologic Research and Information Center (ERIC), VA Puget Sound Health Care System, 1660 S. Columbian Way, S-152-E, Seattle, WA 98108 USA; 2 Rehabilitation Care Services, VA Puget Sound Health Care System, 1660 S. Columbian Way, S-RCS-117, Seattle, WA 98108 USA; 3 Clinical Learning, Evidence, and Research (CLEAR) Center, University of Washington, 4333 Brooklyn Ave NE, Box 359455, Seattle, WA 98104 USA; 4 Department of Rehabilitation Medicine, University of Washington, 325 Ninth Avenue, Box 359612, Seattle, WA 98104 USA; 5 School of Health and Rehabilitation Sciences, The University of Queensland, 84a Services Rd, St Lucia QLD 4067, Brisbane, QLD Australia; 6 School of Public Health, The University of Sydney, A27 Fisher Rd NSW 2006, Sydney, NSW Australia; 7 School of Psychology, The University of Queensland, Sir Fred Schonell Dr, St Lucia QLD 4072, Brisbane, QLD Australia; 8 Department of Biostatistics, University of Washington, 1705 NE Pacific Street, Box 357232, Seattle, WA 98104 USA

**Keywords:** Low back pain, Flares, Flare-up, Episodes, Exacerbations, Recurrence, Disability

## Abstract

**Background:**

Although it is generally accepted that physical activity and flares of low back pain (LBP) are related, evidence for the directionality of this association is mixed. The Flares of Low back pain with Activity Research Study (FLAReS) takes a novel approach to distinguish the short-term effects of specific physical activities on LBP flares from the cumulative effects of such activities, by conducting a longitudinal case-crossover study nested within a cohort study. The first aim is to estimate the short-term effects (≤ 24 h) of specific physical activities on LBP flares among Veterans in primary care in the Veterans Affairs healthcare system. The second aim is to estimate the cumulative effects of specific activities on LBP-related functional limitations at 1-year follow-up.

**Methods:**

Up to 550 adults of working age (18—65 years) seen for LBP in primary care complete up to 36 “Scheduled” surveys over 1-year follow-up, and also complete unscheduled “Flare Window” surveys after the onset of new flares. Each survey asks about current flares and other factors associated with LBP. Surveys also inquire about activity exposures over the 24 h, and 2 h, prior to the time of survey completion (during non-flare periods) or prior to the time of flare onset (during flares). Other questions evaluate the number, intensity, duration, and/or other characteristics of activity exposures. Other exposures include factors related to mood, lifestyle, exercise, concurrent treatments, and injuries. Some participants wear actigraphy devices for weeks 1–4 of the study. The first aim will examine associations between 10 specific activity categories and participant-reported flares over 1-year follow-up. The second aim will examine associations between the frequency of exposure to 10 activity categories over weeks 1–4 of follow-up and long-term functional limitations at 12 months. All analyses will use a biopsychosocial framework accounting for potential confounders and effect modifiers.

**Discussion:**

FLAReS will provide empirically derived estimates of both the short-term and cumulative effects of specific physical activities for Veterans with LBP, helping to better understand the role of physical activities in those with LBP.

**Trial Registration:**

ClinicalTrials.gov NCT04828330, registered April 2, 2021.

## Background

Low back pain (LBP) is the leading cause of years lived with disability worldwide [1] and second only to hearing conditions as a cause of disability among military Veterans in the United States of America (USA) [2]. Minimizing the functional impact of LBP at the population level by modifying risk factors has long been a goal of clinical care and motivates research. Although substantial research has examined risk factors for (1) new episodes of LBP and (2) the acute-to-chronic LBP transition, these commonly studied transitions in health states reflect only two of the numerous possible presentations reflecting symptom worsening in LBP [3, 4]. Other examples of relevant transitions in LBP include the change from low to moderate pain intensity, the change from moderate to high pain intensity, stable pain intensity in the setting of a compensatory strategy (e.g. adaptive coping skills, social support, concurrent treatment) that fails or is no longer available, an increased frequency of episodes with intercurrent symptom-free periods, and various others [4–6]. The identification of risk factors for symptom worsening in LBP may offer new targets for intervention that can help to mitigate both the individual-level and societal-level impact of LBP.

A common manifestation of LBP variability is a “flare” or a “flare-up.” [4] Flares of LBP are periods of transient worsening of symptoms which can reflect an increase in pain intensity or other factors associated with pain such as impaired physical function or changes in mood [6]. Individuals with LBP often attribute flares to recent physical activities [7, 8]. Although it is generally accepted that physical activity and flares of LBP are related, the evidence for the directionality of these associations is mixed. Randomized controlled trials (RCT) of participants with LBP have shown protective effects of physical activity and advice to remain active as demonstrated by improvements in return to work (RTW) and functional limitations [9–11]. On the other hand, studies of occupational activities have had contradictory results, with some activities having deleterious effects on LBP, and differences in activity-LBP relationships depending on the type of activity [12]. Some have suggested non-linear relationships between activity and LBP, whereby activity is beneficial for LBP at moderate levels but not at the extremes of activity [13].

A possible explanation for differing perceptions of the nature of the activity-LBP relationship is that the *transient* (short-term) effects of specific physical activities may differ from the *cumulative* (overall) effects of these activities. For example, a single session of trunk conditioning might increase the chances of a flare of LBP in the short-term, but if performed several times a week over a period of months, trunk conditioning may protect against LBP flares. Such a relationship is analogous to the effect of activity on risk of myocardial infarction—activity confers a detrimental transient effect on myocardial infarction risk, alongside a beneficial cumulative effect [14]. Transient vs. cumulative effects on LBP may vary depending on the specific activity involved. Cumulative effects can be estimated by conventional study designs such as cohort studies. However, transient effects on an outcome that occur within a short period of time after an exposure (e.g., ≤ 24 h) require alternative measurement and analysis approaches [14].

Although clinical guidelines recommend that individuals with LBP should avoid bed rest and try to normalize activities [15–17], they offer no *specific* recommendations about the types of activity (e.g., lifting, bending, etc.) that should be engaged in or avoided *(“Which?”)*, the optimal duration or intensity of such activities *(“How?”*), or the timing of reintroducing such activities *(“When?”*). The guidelines therefore do not address a major concern of individuals with LBP, which is that certain activities may have transient effects on LBP flares [7] or cause sustained detrimental effects on pain or function [18]. In the absence of specific advice, individuals with LBP may experience flares with one type of activity, and then subsequently choose the detrimental path of avoiding all activities [19], as exemplified by one patient testimonial: “Doctors tell you to exercise…but it is difficult to do with back pain. Exercise makes my pain worse, so I just don’t do it.” [20]

The distinctions between beneficial, benign, and detrimental activities are also important to primary care providers (PCPs), who are often called upon to detail recommended limitations for common work-related activities, as part of the RTW process. LBP is the most common reason for work-related claims [21]. Although the specific content of work restriction documentation varies between different worker’s compensation systems at the local, state, and federal levels, all forms typically include certain activities that occur commonly in the workplace or other aspects of everyday life, such as lifting, bending, standing, walking, sitting, etc. An ideal set of work restrictions for LBP would limit those activities that are likely to cause poor overall functional outcomes in the long-term (cumulative effects), whereas decisions regarding return to activities that lead to flares of pain (transient effects) without compromising long-term outcomes may depend on other factors, such as patients’ goals, beliefs about pain, and job descriptions, among other factors. Currently there are no empirically-derived risk estimates which contrast the transient vs. cumulative effects of specific activities for people with LBP, with which to guide work restrictions**.** In the absence of empirical data, work restrictions by PCPs mirror their general practice style [22, 23] or personal beliefs [22], which can conflict with evidence-based care [24]. Moreover, PCPs often feel pressured to choose work restrictions that avoid conflict with their patients [23, 25, 26]. These factors combine to create a situation where most PCPs feel ill-prepared or conflicted when making work restriction recommendations [27]. Objective data are needed regarding the short- and long-term risks associated with specific types of activities during LBP, in order to meet an important need of patients and PCPs.

In addition to physical activities, it is also important to consider other time-varying exposures that may affect LBP outcomes. These include the well-studied psychological factors of depression, stress, catastrophizing, fear of movement, coping skills, self-efficacy, acceptance, and beliefs about the underlying nature of LBP [28–30]. Post-traumatic stress disorder (PTSD) has also been shown to predict LBP outcomes in longitudinal studies of Veterans [31]. Lifestyle related factors such as sleep quality, smoking, alcohol consumption, other substance use, and leisure-time exercise also may affect LBP outcomes [32]. Additionally, individuals with LBP commonly use non-pharmacologic and pharmacologic treatments to manage LBP symptoms, and these treatments may affect or confound LBP outcomes in the short- and/or long-term.

We propose a novel approach to distinguish the transient effects of specific physical activities on LBP flares from the cumulative effects of such activities, while accounting for a wide range of other time-varying exposures which may have independent effects on LBP, by conducting a longitudinal case-crossover study nested within a cohort study. The case-crossover design accounts for individual-level measured and unmeasured confounding by using each participant as their own control, analogous to a crossover experiment. The first aim of this study is to estimate the *transient* effects (≤ 24 h) of specific physical activities on flares of LBP among Veterans in primary care in the Veterans Affairs (VA) healthcare system. The second aim of this study is to estimate the *cumulative* effects of specific physical activities on LBP-related functional limitations at 1-year follow-up.

## Methods/design

This protocol uses the term “effect” throughout when referring to the relationships between exposures and LBP outcomes (flares or back-related functional limitations), in order to remain consistent with the terminology used in the case-crossover literature [33]. Therefore, all uses of the term “effect” here pertain to conclusions made from analyses of observational data, rather than that from an RCT, which is the typical standard used to establish causal effects in clinical research. However, most of the transient, time-varying exposures examined in the current study— such as specific physical activities— do not lend themselves to randomized designs, leaving observational approaches as the most rigorous option for making causal inferences about these factors.

### Terminology

Table 1 presents terminology pertaining to LBP flares and the case-crossover design as used in the study described by this protocol. As described above, LBP “flares” are a period of transient worsening of symptoms which can reflect an increase in pain intensity or a deterioration in other factors associated with pain, such as physical function or mood [6]. The terms “episodes” and “recurrences” refer to periods of LBP preceded by a symptom-free period, and may be viewed as more specific instances of flares [34, 35].

**Table 1:** Terminology pertaining to the effects of activity during LBP

**Term**	**Definition**
**Flare**	An increase in pain (such as LBP), typically lasting hours to days [7]. Flares may occur either in the setting of existing pain, or in the setting of minimal LBP or no LBP [6]
**Episode**	A specific instance of a flare, in which LBP is preceded by a period of minimal or no LBP
**Recurrence**	A new episode of LBP, preceded by a period of minimal or no LBP, preceded by a past history of LBP [6, 35, 36]
**Trigger**	An exposure with a transient effect. Triggers may often have transient durations [33] *Example: Lifting may ‘trigger’ the abrupt onset of a flare of LBP*
**Transient effect (short-term effect)**	An effect on an outcome that occurs within a short period of time (e.g., ≤ 24 h) after an exposure, such as estimated in a case-crossover study [33] *Example: Lifting a heavy weight might cause an immediate flare of LBP*
**Long-term effect**	An effect on an outcome that occurs over longer periods of time (e.g. days to weeks)
Cumulative effect (overall effect)*	An *overall* effect within which both transient effects and long-term effects are subsumed. Cumulative effects are estimated by cohort studies and RCTs [37] *Example: Lifting heavy weights often may increase functional limitations at 12-month follow-up*
**Effect period**	The duration within which the transient effect of an exposure can be expected to manifest, *in a target population *[33]. The effect period of activities on LBP is thought to be < 24 h [7, 38]

*LBP* low back pain, *RCT* randomized controlled trial

In clinical practice, individuals with LBP often perceive the effects of an activity exposure on LBP manifesting at or soon after an activity, or on the day after the activity [7]. Consistent with this, a longitudinal study determined the effect period of activity on LBP to be ≤ 24 h [38]. A 24 h effect period is also supported by the results of a pilot study conducted in preparation for the current work [7]. In contrast, an earlier retrospective study assumed a shorter (< 2 h) effect period of activities [39]. Taken together, prior work suggests 24 h as a plausible effect period of activity, but a 2 h effect period (subsumed within the 24 h period) may also warrant study. The proposed methods in the current study will obtain information on both the 24 h *and* 2 h effect periods of self-reported activity exposures, permitting a comparison of the two and a determination of which is most appropriate.

### The case-crossover study design

**Fig. 1:**
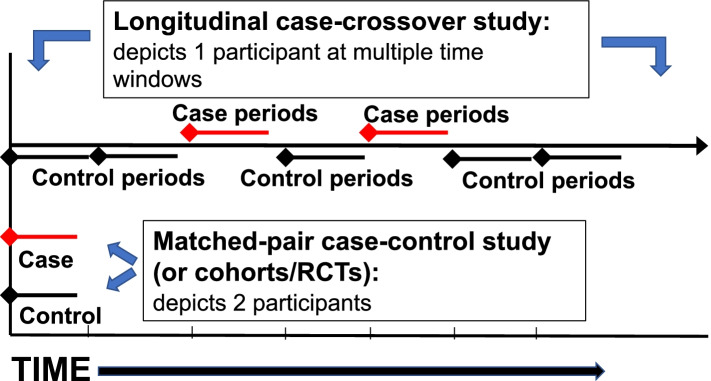
The longitudinal case-crossover design

Effects of an exposure on an outcome may be either transient (e.g., effects on pain flares) or long-term (i.e., sustained effects) (Table 1). Cohort studies and RCTs estimate *cumulative effects*, which are a weighted mixture of transient and long-term effects [37]. These designs compare exposure status in case *persons* with exposure status to control *persons* in order to make inferences about risks (Fig. 1). The case-crossover design was developed specifically to study the transient effects of time-varying exposures [33], and when nested within a cohort study, the design can help to distinguish the transient effects of exposures from the cumulative effects of such exposures. The case-crossover design compares the exposure status during ‘case windows’ of *person-time* with the exposure status during “control windows” of *person-time* (Fig. 1); within-person estimates of risk can be pooled across persons to make inferences concerning the transient risks associated with transient exposures. A case-crossover study is therefore analogous to a case–control study, but the former uses control *times* as the referent group whereas the latter uses control *persons*. By having each person serve as their own control, the case-crossover design has the major advantage of eliminating confounding by person-level factors that are fixed or relatively stable over time [33, 37]. Although early case-crossover studies of LBP assessed case and control windows retrospectively [39], incurring potential recall bias, such bias should be mitigated by using the prospective, longitudinal case-crossover design.

### Ethical approval

The Institutional Review Boards at the VA Puget Sound Health Care System (VAPSHCS) and the University of Washington (UW) approved the study and all processes for informed consent. There are separate informed consent processes at each institution for the components of the project conducted at VAPSHCS, and the components of the project conducted at UW. At VAPSHCS, at the start of the study, all informed consent was written informed consent. Subsequently, Institutional Review Board approval was obtained to provide an Information Statement to potential participants and a summary of both VAPSHCS and UW components of the project. The Information Statement is verbally reviewed with the potential participant, and verbal informed consent is obtained. At UW, an electronic Information Statement is provided to potential participants, and participants indicate their acknowledgement of the Information Statement and consent to participate in the study electronically.

### Study participants

The target population is Veterans seen for LBP in primary care at VAPSHCS. The VA is the largest integrated healthcare system in the US. VAPSHCS provides health care to Veterans in a five-state region (Washington, Alaska, Montana, Idaho, and Oregon), but most enrollees reside in western Washington state. Veterans of working age (18—65 years) who are seen for LBP in primary care at VAPSHCS will be invited to take part in the study. LBP is defined as pain in the area of the low back between the lowest ribs and the gluteal crease, irrespective of leg pain and radiating symptoms. Inclusion criteria are having regular access to technology and the Internet sufficient to complete frequent electronic surveys; basic computer literacy; and being able to understand and read English. Potential participants are excluded if they have a “red flag” condition (diagnoses indicative of a specific medical cause of pain, such as malignancy, infection, etc.); are pregnant; are an incarcerated person; have a severe medical or psychiatric comorbidity likely to be a barrier to study completion (e.g. a terminal medical condition); have a primary psychotic or major thought disorder; being hospitalized for psychiatric reasons involving psychosis in the past 5 years; have a diagnosis indicating cognitive limitations which would limit study participation; have had thoracolumbar spine surgery in the past 1 year; or have planned major orthopedic, abdominal, or chest surgery in the next 2 months.

### Recruitment

Up to 550 participants will be recruited via two modes: (1) in-person recruitment at the time of primary care clinic visits for LBP, and (2) remote recruitment by mail and telephone. Due to COVID-19, a decision was made prior to the start of the study to limit recruitment to remote recruitment by mail and telephone until public health conditions permitted in-person recruitment with minimal risks to patients and research staff. At the time of this writing, all recruitment has been conducted remotely (mode 2).

#### Mode 1 (in-person recruitment)

Research staff will approach Veterans seeking care for LBP in primary care directly after an in-person clinical encounter. The study will be explained and informed consent will be offered.

#### Mode 2 (mail and telephone-based recruitment)

On a recurring basis, research study staff identify potentially eligible patients seen in primary care, using ICD-10 codes indicating LBP or related spinal disorders [40] obtained from centralized administrative data sources reflecting all clinical care provided at VAPSHCS. Potentially eligible Veterans are mailed information packets which prompt the patient to contact research staff by telephone if they are interested in the study. Recruitment procedures and the informed consent discussion are administered over the phone.

During the informed consent process and after consent has been obtained, participants receive education from a research assistant regarding the study design, how to register in the web portal, and how to complete e-Surveys. Participants also receive printed materials containing links, screenshots, and detailed instructions regarding these aspects of participation. Participants are encouraged to contact study staff with questions or problems at any point in the study.

### Flare definition

Prior to the start of data collection, participants are provided information regarding the flare definitions used in the current study. A “flare” is defined as “*a worsening of your low back pain that lasts from hours to weeks*, but no longer than a month”. Other statements explain what qualifies as a flare for the purposes of the study, including: “A slight change in your low back pain intensity does not count as a flare” (to ensure that trivial increases in pain are not reported as flares); “A worsening of your low back pain that lasts seconds to minutes does not count as a flare, because it is too short” (to ensure that momentary increases in pain are not reported as flares); and “A worsening of your low back pain that lasts 1 month or more also does not count as a flare, because it is too long” (as after 1 month at a higher level of pain intensity, patient can be considered to be at a new [higher] baseline level of pain). This information is also available as a reference on the participant portal throughout the study.

### Data collection

Data sources include electronic surveys (“e-Surveys”) completed by participants using a secure web-based portal, actigraphy, and the electronic health record (EHR). There are 4 types of e-Surveys: (1) Baseline e-Surveys, (2) Scheduled e-Surveys, (3) Flare Window e-Surveys, and (4) Exit e-Surveys. Participants complete e-Surveys using their own personal electronic devices (smartphones, tablets, or personal computers, not provided by the study). Tables 2 and 3 contain descriptions of the study measures assessed by participant self-report, including citations which provide further information on the measures.

**Table 2:** Baseline and exit E-survey questionnaires

**Domain**	**Measure or description**
**Low Back Pain (LBP) Characteristics**	
LBP history	LBP in last 24 h, duration of LBP, frequency of LBP in last 6 months [41]
LBP intensity	0–10 LBP NRS in the last 24-h: average pain, and worst pain [41]
LBP trajectory	Visual Trajectories Questionnaire for Pain (VTQ-P) [42]
Back-related functional limitations	Self-report changes in back pain in the last year: Roland-Morris Disability Questionnaire [43]
Overall global health	PROMIS Global Health Short Form v1.2: 10-item measure [44]
Pain homunculi	Pain manikin with body regions, neck pain, mid/upper back pain, in last 12 months
Back surgery questions	Self-reported history of back surgery, and number of back surgeries [41]
Past LBP treatments	LBP treatments (e.g., physical therapy, spinal manipulation, acupuncture, etc.) received for LBP: ever and/or in the past 1-year [41]
Analgesic use	Analgesic use in the last 8-h, common analgesic medication categories
Sleep duration/quality	Typical time going to sleep and waking on weekday and weekend [45]; PROMIS Sleep Disturbance [46]
**Physical activity**	
General	Self-reported average minutes per week of moderate intensity activity, and vigorous intensity activity [47, 48]
Specific physical activities and perceived effects on LBP	Self-reported perception of activities’ effects on LBP (e.g., lifting or lowering, carrying, pushing or pulling, sitting, standing, walking, bending or stooping, twisting, squatting or kneeling, crawling)
Substance use	Self-reported frequency of use of non-illicit substances (e.g., cigarettes, e-cigarettes, vaporizers, alcohol) [41, 49]
**Psychological Factors**	
Depression	PROMIS Depression Short form v.1.0 8b: single-item depression measure [50]
PTSD	Post-Traumatic Stress Disorder Checklist-Civilian Version (PCL-C) [51]
Catastrophizing	UW Concerns about Pain Scale 8-item short form (UW CAP-8) [52]; Coping Strategies Questionnaire (CSQ) 2-item version [53]
Fear of movement	Tampa Scale of Kinesiophobia [54]
Acceptance	Chronic Pain Acceptance Questionnaire (CPAQ-8) [55]
Self-efficacy	UW Pain-Related Self-Efficacy Scale 6-item short form (UW PRSE-6) [56]^a^
Back-related pain beliefs	Back Pain Attitudes Questionnaire (Back PAQ) 10-item version [57]
Global rating of change	Self-reported changes in back pain in last 1-year^b^
Sociodemographic	Age, sex, gender identity, race, ethnicity, marital status, height, weight, education, current employment, income^a^
**Work**	
Occupation	Self-report of occupation and work industry [58]
Typical frequency of specific physical activity categories at work	Lifting/lowering 10 + pounds, Lifting/lowering 25 + pounds, carrying, pushing/pulling, sitting, standing, walking, bending/stooping, twisting, squatting, crawling
Work productivity	Work Productivity and Activity Impairment 6-item measure [59]
Other work-related factors	Borg scale of physical demands at work [60], Andrews and Withey Job Satisfaction Questionnaire [61]^a^

*Back PAQ* Back Pain Questionnaire, *CAP-8* UW Concerns about Pain Scale 8-item short form, *CPAQ-8* Chronic Pain Acceptance Questionnaire, *CSQ* Coping strategies Questionnaire, *LBP* Low back pain, *NRS* Numerical rating scale, *PCL-C* Post-Traumatic Stress Disorder Checklist-Civilian Version, *PROMIS* Patient Reported Outcome Measurement Information System, *VTQ-P* Visual Trajectories Questionnaire for Pain, *UW PRSE* University of Washington Pain-Related Self-Efficacy Scale

^a^Administered during the Baseline e-Survey only

^b^Administered during the Exit e-Survey only

**Table 3:** Scheduled and flare window E-survey questionnaires

**Domain**	**Measure or description**
**Flares, low back pain (LBP) intensity, and pain interference**	
Flare characteristics	Flare-up at present (“Are you currently experiencing a flare of your low back pain?”), date of onset, continuation since last reported flare, duration of current flare, any unreported flares since last survey
Pain intensity	LBP intensity, right now; worst LBP intensity in past 24 h [41]
Analgesic use	Analgesic use in the last 8-h, common analgesic medication categories
General activity interference	LBP interference with general activity, right now [62]
**Psychological factors**	
Depression	Single-item depression measure [50]
PTSD	Primary care-PTSD screen (PC-PTSD) [63]
Stress	Single-item stress scale [64, 65]
Catastrophizing	Coping strategies questionnaire (CSQ) 2-item version [53]
Fear of movement	Tampa Scale of Kinesiophobia (TSK) 2-item version; [54]
Self-efficacy	UW Pain-Related Self-Efficacy Scale short form 2-item (UW-PRSE-2) [56]
Work	Work Productivity and Activity Impairment 4-item measure [59]
**Primary Physical Activity Categories (10 major types)**	
Lifting or Lowering	For each increment of weight lifted/lowered (10–19 lbs., 20–29 lbs., 30 + lbs.), participants report frequency, duration, concurrent actions (e.g., twisting, bending down, carrying, etc.), avoidance of the activity, and reasons for avoidance. Other questions pertain to maximum weights lifted and frequency/duration of maximum weights lifted
Pushing or Pulling	Frequency, typical weight pushed or pulled, maximum weight pushed or pulled, avoidance of pushing or pulling, and reasons for avoidance
Sitting, Standing, Walking	Total duration of each activity, duration of continuous activity, setting of activity, avoidance of the activity, and reasons for avoidance
Other Activity Categories	Frequency of each activity (e.g., bending or stooping, climbing, twisting, squatting or kneeling, crawling): duration, avoidance of the activity, and reasons for avoidance
Sports and Exercise	Participation in endurance exercise (e.g., running, biking, etc.), low impact exercise (e.g., walking for exercise, yoga, etc.), sports (e.g., baseball, racquet sports, martial arts, etc.), weightlifting, dancing, etc. [66] Other questions pertain to avoidance of sports/exercise and reasons for avoidance.
**Other Exposures**	
Household or Outdoor Activities	Recent household or outdoor activities, including endurance common household activities (e.g., cleaning, laundry, painting, etc.), outdoor non-sport activities (e.g., carpentry, construction, farming, etc.). Other questions inquire about avoidance of activity, reason for avoidance [67, 68]
LBP Treatments	Physical treatments (e.g., strengthening exercises, stretching exercises, etc.), modalities (e.g., heat, ice, acupuncture, etc.), behavioral treatments (e.g., individual/group therapy, support groups, etc.), procedures
Injuries	Fall, near falls, landing from a jump, slipping, tripping, motor vehicle accidents
Sleep	Duration of actual hours slept in last 24-h, quality of sleep in last 24-h [69, 70]
Substance use	Self-reported frequency of use of non-illicit substances (e.g., cigarettes, e-cigarettes or vaporizers, alcohol) [41, 49]
Fatigue Severity	PROMIS fatigue item [71]

*CSQ* Coping strategies Questionnaire, *PC-PTSD* Primary Care- Post-Traumatic Stress Disorder, *PROMIS* Patient Reported Outcome Measurement Information System, *TSK* Tampa Scale for Kinesiophobia, *UW PRSE* UW Pain-Related Self-Efficacy Scale

#### Baseline e-Survey

The Baseline e-Survey asks for information on participants’ history of LBP, LBP intensity and functional limitations, general health status, other locations of pain, past LBP treatments, sleep, physical activities, perceptions of physical activities’ effects on LBP, substance use, psychological factors (depression, PTSD, catastrophizing, fear of movement, acceptance, self-efficacy, and beliefs about LBP), sociodemographics, and work-related factors (disability or compensation status, hours/days off work, job satisfaction rating, physical demands at work) (Table 2).

#### Scheduled e-Surveys

Scheduled e-Surveys are cue-elicited assessments administered 36 times over 1-year follow-up: three times each week during weeks 1–4 of follow-up (phase 1), once a week during weeks 5–8 (phase 2), and twice a month during months 2–12 (phase 3). The specific timing of days when Scheduled e-Surveys are administered was randomly generated using computer software applying several constraints, including accounting for the different sampling frequencies in each phase, minimizing surveys on consecutive days, and allowing sampling on all days of the week. Cues to complete Scheduled e-Surveys are automated “e-Alert” notifications sent by text message and/or email at the start of each “scheduled assessment window,” a 3-h period during which a scheduled e-Survey may be completed. Participants are able to pre-select recurring time slots of any duration each day (e.g., 11am-4 pm, or 7am-9 pm) that are generally most convenient for completing surveys. Participants receive an e-Alert at a random time within their pre-selected time slot and have 3 h from the time of this e-Alert to complete the survey. Participants receive 2 reminder e-Alerts at 1-h intervals after the initial e-Alert.

The first question of each e-Survey asks participants to report whether they are currently experiencing a flare by responding to the question “*Are you currently experiencing a flare of your low back pain?*”. Additional information presented to participants with the first question of each e-Survey reminds them of the flare definitions used in this study, including the content described above in the “Flare Definitions” subsection. Further questions inquire about factors commonly reported as being part of the experience of having a flare, including whether the current flare has been difficult to tolerate, has impacted usual activities, has impacted emotions, has required a new pain medication or more pain medications than usual to manage, or has required a new treatment (other than a patient’s routine treatments) to manage [6]. Other questions inquire about current pain intensity and pain interference (Table 3).

Participants are asked about recent exposures to 10 major types of physical activities (“activity categories”) that are commonly asked about in work restriction documentation, including lifting or lowering, pushing or pulling, sitting, standing, walking, bending or stooping, climbing, twisting, squatting or kneeling, and crawling. Additional modules inquire about other recent exposures of interest, including psychological factors, physical activities, LBP treatments, injuries, sleep, substance use, and fatigue assessed using brief, validated measures (Table 3). If a participant reports not currently experiencing a flare (i.e., a “non-flare” period) at the beginning of an e-Survey, subsequent questions in a survey ask about exposures of interest during the 24 h *prior to survey completion*, and the 2 h prior to survey completion. In this way, Scheduled e-Surveys are inherently time lagged for exposure-flare relationships, as they capture current flare status at time = t, and activities in the 24-h period (and 2-h period) before the start of the assessment window (t-1). If a participant reports currently experiencing a flare (i.e., a “flare period”), subsequent questions ask about exposures of interest during the 24 h *prior to flare onset*, and the 2 h prior to flare onset. Scheduled e-Surveys when a flare is reported therefore capture current flare status at time = t, and activities in the 24-h period (and 2-h period) before flare onset (t-1).

Because of the high burden of study participation, a run-in period is used to enrich the sample for participants capable of completing frequent cue-elicited surveys with high response rates and exclude those who are unlikely to provide sufficient data for the planned analyses. Participants are expected to complete 4 of the first 6 Scheduled e-Surveys during the first 2 weeks of participation. Allowances are made for reasonable factors that affect adherence during the run-in period (e.g., life events such as medical problems, technological barriers, travel, etc.). Participants who do not pass the run-in period are withdrawn from the study.

#### Flare Window e-Survey

Flare Window e-Surveys are available on the study portal for participants to complete on an *ad hoc* basis if they experience a new flare of LBP at any time during 1-year follow-up. Participants are educated on the need to initiate a Flare Window e-Survey within 3 h of flare onset, a design feature intended to minimize recall bias. The content of Flare Window e-Surveys is the same as that of the Scheduled e-Surveys (Table 3), with the recall period for exposures specified as the period prior to flare onset.

#### Exit e-Survey

The Exit e-Survey becomes available on the web portal at the end of the 1-year follow-up period. The Exit e-Survey includes most of the measures that comprised the Baseline e-Survey, as well as measures of global perceived improvement and satisfaction (Table 2).

#### Actigraphy

Participants are invited to participate in an optional portion of the study that involves wearing an actigraphy device (ActiGraph wGT3X-BT) for weeks 1–4 of the study for objective assessment of physical activity. The device is worn continuously on the thigh using a specialized thigh strap, except for when in contact with water (e.g., showering, bathing, swimming). Participants are provided with a document outlining instructions for how to use and wear the device, how to care for the device, and instructions on how to return the device. ActiGraph data are extracted using proprietary software and will be used to evaluate the number and frequency of walking steps each day, time spent sitting, and sleep/wake data.

#### Electronic Health Record (EHR) data

EHR and administrative data are collected for all participants, including sociodemographics, pharmacy data, laboratory data, vital signs, diagnostic codes, procedure codes, and problem lists. This includes all available EHR data prior to recruitment, during the 1-year follow-up period, and up to 2 years after the time of recruitment. EHR data will be used to characterize the study sample with respect to LBP history and use of healthcare services, and to identify clinical subgroups that will be accounted for in secondary analyses.

### Statistical analyses

#### Aim 1 analyses

We will examine associations between activity category exposures and the participant-reported flares outcome in conditional logistic regression models, using data from all Scheduled and Flare window e-Surveys over 1-year. There will be separate models for each of the 10 primary activity categories. Models will include activity categories as time-varying predictors as in Eq. 1, where *Y* is the outcome (flare vs. no flare), *x* is the activity exposure (yes vs. no), and C represents potential time-varying confounders (psychological factors, other activities, etc.). The letter *i* indicates the *i*^th^ individual (*i* = 1,2,... n), *j* indicates the *j*^th^ time of follow-up (*j* = 1,2,...,n_*i*_) and *x*_*i(j-1)*_ indicates an activity that was performed in the exposure window prior to the *j*^th^ time either the start of the control window (Scheduled e-Survey) or time of flare onset (Flare Window e-Survey). As previously noted, the survey methods are such that activity exposure and flare outcome are inherently time-lagged, with exposure preceding outcome. β_w_ represents the association of activity *x* with flare outcome Y within individuals (the “within-individual association”), or the expected change in the odds of a flare with change in the activity exposure for a given individual. These analyses will yield odds ratios (OR) and 95% confidence intervals for within-individual “effects” of activity triggers on an LBP flare. The initial analytic approach will examine the association between binary activity exposure and binary flare outcome, with explicit adjustment for the numerical rating scale (NRS) of LBP intensity and number of prior flares measured at time = *j*-1, and time-variant confounders selected based on conceptual importance. By design, the case-crossover method uses each participant as their own control by comparing case periods to control periods within the same participant. This approach accounts for known and unknown confounders that are fixed or relatively stable over time (i.e., age, sex, sociodemographics, medical and LBP history, work-related factors, and underlying psychological predispositions). We will use a Bonferroni-corrected threshold of *p* = 0.005 for each primary activity-flare comparison to account for 10 statistical comparisons (one for each of the 10 activity categories). We will also consider alternate methods to conditional logistic regression, such as mixed-effects logistic regression, which allows estimation of both within-individual and “between-individual associations”. We will further examine activity-flare associations after accounting for important relationships based on theory and clinical knowledge, including dose–response relationships between activities and flares and different effect periods of activity exposures (i.e., 24 vs. 2 h), and possible biases (e.g., conscious activity avoidance and carry-over effects). We will conduct multivariable analyses including other potential confounders (other time-varying activities, depression, PTSD, stress, catastrophizing), with careful consideration regarding factors which may be mediators. We will examine possible effect modifiers, including whether activities were work-related; pain intensity; duration of LBP; analgesic use and other treatments; beliefs about LBP and activity-LBP relationships at baseline; and clinical subgroup analyses (e.g., lumbosacral radicular syndrome [LSRS], symptomatic lumbar spinal stenosis [SLSS] diagnosed by a specialist, or lumbar facet-mediated pain as diagnosed by response to lumbar radiofrequency ablation of the medial branch nerves).

1 $$log\left[\frac{\mathrm{Pr}({Y}_{ij}=1)}{1-\mathrm{ Pr}({Y}_{ij}=1)}\right]= {\beta }_{0i}+{\beta }_{w}{x}_{i(j-1)}+\gamma {C}_{i(j-i)}, i=\mathrm{1,2},\dots ,n;j=\mathrm{1,2},\dots ,{n}_{i}$$

After these analyses using the binary flare outcome, we will use an analogous approach to examine relationships between activity categories and the secondary (continuous) outcome of NRS LBP intensity, using linear mixed-effects regression. These analyses may mitigate potential biases that can be introduced by participant self-identification of flare status; if no such bias exists, we would expect the direction of effects to be similar to those obtained in the primary analysis of the binary flare outcomes [72, 73]. We will use analogous methods to analyze ActiGraph data reflecting walking, standing, and sitting. Of note, analyses of walking, standing, and sitting using the ActiGraph data will examine associations in the weeks 1–4 of follow-up only, as this is the only period during which participants will wear the ActiGraphs.

#### Aim 2 analyses

We will examine associations between the frequency of activity during e-Surveys (% of periods during which each of the 10 primary activity categories were reported over the first 8 weeks of follow-up), and the outcome of long-term LBP-related functional limitations at 1 year as reflected by the Roland-Morris Disability Questionnaire (RMDQ) [43], using a Bonferroni-corrected threshold of *p* = 0.005. The percentage of periods with each activity category over weeks 1–8 will be calculated from self-reported activity information, and time spent walking, standing, and sitting over the weeks 1–4 will be estimated using the ActiGraph data. The main multivariable model for each activity category will explicitly adjust for a range of potential confounders based on prior knowledge and conceptual rationale including baseline RMDQ score, psychological predictors, and other baseline covariates from Table 2. We will then follow an analytic approach analogous to that done for Aim 1 to examine activity-12-month-RMDQ associations after accounting for important relationships based on theory and prior knowledge including: moderation by baseline employment status, compensation status, or job satisfaction; moderation by baseline pain intensity, LBP duration, analgesic use or other treatments, beliefs about back pain, and clinical subgroups (LSRS, SLSS, lumbar facet-mediated pain). We will also use the same approach to examine associations with the secondary outcomes of LBP intensity, lost work productivity, quality of life, and opioid use at 12-months.

#### Interpretation

Aims 1 and 2 will yield tables of risk estimates for (1) the *short-term effects* of the 10 specific activity categories on flares and (2) the *overall effects* of such activities on functional recovery as reflected by the 1-year RMDQ score. We expect that activity categories will have different patterns of short-term vs. overall effects. In instances where short-term and overall effects are in the same direction, potential implications for activity recommendations may be straightforward. For example, if lifting confers both greater short-term risk of pain flares and greater overall risk of functional limitations at 12-month follow-up, the clinical implication may be that lifting should be avoided for those with LBP. In some instances where the short-term vs. overall effects of activities differ, however, the clinical implications may depend on personal priorities and preferences. For example, if walking increases the short-term risk of a flare but has no effect on functional recovery at 12 months, the decision of whether or not to walk during LBP can be guided by a patient’s preferences, work-related factors such as urgency in the patient returning to work, or other factors. In other instances where the short-term vs. overall effects of activities have opposite directions of effect, the latter may take priority (i.e., if walking improves functional recovery at 12 months, walking should likely be encouraged even if it causes flares in the short-term).

#### Sample size calculations

Key determinants of statistical power for the Aim 1 case-crossover analysis include the (1) activity exposure effect size, (2) activity frequencies (expected to be 5–25% based on the pilot [7]), (3) number of case/control periods, and (4) proportion of participants who are statistically informative (with both case and control periods). Given the temporary nature of flares, in our view, only activity exposures with moderate magnitude effects (ORs of 1.5–2.0) will have a meaningful impact on the population level. Informed by the characteristics of activities, flares, and missing data from our pilot [7], we estimated power using simulated data with 1000 replications assuming α = 0.005 (Bonferroni-corrected for 10 activity categories), comparing different assumptions for sample size determinants, including activity category frequencies as low as 3%. A sample size of *n* = 440 yielded ≥ 90% power to detect ORs of 1.5 for activity category frequencies ≥ 10%, 80% power to detect OR = 1.6 at activity category frequency = 5%, and 80% power for OR = 1.8 given activity category frequencies as low as 3%. Allowing for 20% of participants not completing the run-in period or not providing a high number of frequent, serial assessments, this indicates the need for 550 participants in Aim 1. These power estimates are conservative, because (1) they use data from Scheduled e-Surveys only and assume no data from *ad hoc* Flare-Window e-Surveys, and (2) they assume no information from the 110 participants not expected to complete the run-in period. Moreover, they are based on binary activity exposures, and analyses using continuous exposures (incorporating duration and frequency) will have greater power. During project execution, if activity category frequencies are higher than the estimates of 3–10% used in our simulations, expected power will be substantially higher and sample size requirements may be revised downwards. For Aim 2, using the same data simulated for Aim 1, and assuming an expected mean (standard deviation) 12-month RMDQ scores of 8.8 (6.4) from a prior study [74], Bonferroni-corrected α = 0.005, activity frequencies ≤ 20%, and *n* = 440 without missing data (from *n* = 550 initially recruited), we estimated power over a range of scenarios where we varied the strength of the association between the *change* in activity frequency over the weeks 1–8 of follow-up (for each of the 10 activity categories) and a minimum clinically important change in 12-month RMDQ of 2.5 points [43]. We found ≥ 80% power to detect a 2.5-RMDQ-point difference at 12 months in scenarios where a 65% change in activity frequency over the weeks 1–8 produced a 2.5-RMDQ-point difference at 12 months, and in all scenarios where the activity frequency-RMDQ relationships was more extreme (e.g., if a smaller % change in activity frequency produced a 2.5-RMDQ-point difference at 12 months). This indicates adequate power for Aim 2 with the expected sample size of 550 participants. If the sample size is less than 550, Aim 2 will still be powered to detect situations where a < 65% change in activity produces a 2.5-RMDQ-point difference at 12 months.

## Discussion

This will be the first longitudinal case-crossover study of LBP nested within a cohort study that includes long-term follow-up. This design will enable the study to provide the first empirically derived estimates of both the short-term effects and overall effects of specific physical activities for adults with LBP. These findings can be used to provide specific guidance to help support Veterans with LBP in how to best optimize physical activity levels while maintaining or improving their back-related physical function in the long-term. These findings are also likely to translate to non-Veterans. We also expect these findings to assist clinicians in recommending work restrictions during the RTW process. The information produced by this study will inform the future development of educational programs and interventions to optimize physical activities and physical function for Veterans and others with LBP.

Strengths of this study include a large sample size, the longitudinal design, frequent sampling methods using ecological momentary assessment procedures, long-term (1-year) follow-up, and the collection of information on a wide range of covariates which will control for many potential confounders. The design also includes a flare-elicited component of data collection designed to capture periods of symptom worsening soon after the onset of such periods, improving participants’ recall of recent exposures and making recalled periods more comparable between periods preceding a flare and periods not preceding a flare. On the other hand, this design feature may not entirely protect against bias induced by differences in how participants remember recent exposures depending on their current pain status (e.g., a person who believes a type of activity causes their flares may selectively remember recent activity exposures of that type immediately after a flare but tend to forget recent activity exposures of that type when not having a flare). To account for this, we will conduct secondary analyses accounting for participants’ beliefs about specific activity-LBP relationships at baseline. Moreover, for some activity types (e.g., walking), we will be able to verify findings regarding activity-LBP relationships using objectively-measured actigraphy-assessed measures. A limitation of the study is the self-reported nature of physical activities in our study. While partially mitigated by the use of actigraphy, current technology makes it infeasible or impossible to accurately and objectively measure some activities with possible links to LBP flares (e.g., lifting, twisting, crawling) in real-world or “free-living” situations over longer periods of time, leaving no viable alternative to self-report.

The FLAReS study will create a rich longitudinal database to better understand the role of physical activities for Veterans with LBP. This resource will allow completion of main research aims as described in this protocol, and will also be a valuable resource for ongoing research.

## Data Availability

Data sharing is not applicable as no datasets were generated or analyzed as part of the study protocol.

## Abbreviations

Back PAQ: Back Pain Questionnaire
CAP-8: UW Concerns about Pain Scale 8-item short form
CCCR: Core Center for Clinical Research
CLEAR: University of Washington Clinical Learning, Evidence, and Resarch
COVID-19: Coronavirus Disease 2019
CPAQ-8: Chronic Pain Acceptance Questionnaire
CSQ: Coping strategies Questionnaire
EHR: Electronic Health Record
FLAReS: Flares of Low back pain with Activity Research Study
ICD: International classification of diseases
LBP: Low back pain
LSRS: Lumbosacral radicular syndrome
NIAMS: National Institute of Arthritis and Musculoskeletal and Skin Diseases
NRS: Numerical rating scale
OR: Odds ratio
ORD: Office of Research and Development
PC: Personal Computer
PC-PTSD: Primary Care- Post-Traumatic Stress Disorder
PCL-C: Post-Traumatic Stress Disorder Checklist-Civilian Version
PCP: Primary care provider
PTSD: Post-traumatic stress disorder
PROMIS: Patient Reported Outcome Measurement Information System
RCT: Randomized controlled trial
RMDQ: Roland Morris Disability Questionnaire
RR&D: Rehabilitation Research and Development
RTW: Return to work
SLSS: Symptomatic lumbar spinal stenosis
T: Time
TSK: Tampa Scale for Kinesiophobia
USA: United States of America
UW: University of Washington
VA: Veterans Affairs
VAPSHCS: VA Puget Sound Health Care System
VTQ-P: Visual Trajectories Questionnaire for Pain
UW PRSE: UW Pain-Related Self-Efficacy Scale
